# Metabonomic Profiling of Serum and Urine by ^1^H NMR-Based Spectroscopy Discriminates Patients with Chronic Obstructive Pulmonary Disease and Healthy Individuals

**DOI:** 10.1371/journal.pone.0065675

**Published:** 2013-06-06

**Authors:** Lingling Wang, Yufu Tang, Shuo Liu, Shitao Mao, Yuan Ling, Dan Liu, Xiaoyu He, Xiaoge Wang

**Affiliations:** 1 Department of Respiratory Medicine, The Fourth Affiliated Hospital of China Medical University, Shenyang, People’s Republic of China; 2 Department of General Surgery, 202 Hospital of Chinese PLA, Shenyang, People’s Republic of China; Instituto de Investigación Sanitaria INCLIVA, Spain

## Abstract

Chronic obstructive pulmonary disease (COPD) has seriously impacted the health of individuals and populations. In this study, proton nuclear magnetic resonance (^1^H NMR)-based metabonomics combined with multivariate pattern recognition analysis was applied to investigate the metabolic signatures of patients with COPD. Serum and urine samples were collected from COPD patients (n = 32) and healthy controls (n = 21), respectively. Samples were analyzed by high resolution ^1^H NMR (600 MHz), and the obtained spectral profiles were then subjected to multivariate data analysis. Consistent metabolic differences have been found in serum as well as in urine samples from COPD patients and healthy controls. Compared to healthy controls, COPD patients displayed decreased lipoprotein and amino acids, including branched-chain amino acids (BCAAs), and increased glycerolphosphocholine in serum. Moreover, metabolic differences in urine were more significant than in serum. Decreased urinary 1-methylnicotinamide, creatinine and lactate have been discovered in COPD patients in comparison with healthy controls. Conversely, acetate, ketone bodies, carnosine, m-hydroxyphenylacetate, phenylacetyglycine, pyruvate and α-ketoglutarate exhibited enhanced expression levels in COPD patients relative to healthy subjects. Our results illustrate the potential application of NMR-based metabonomics in early diagnosis and understanding the mechanisms of COPD.

## Introduction

Chronic obstructive pulmonary disease (COPD) is one of the most common chronic adult respiratory diseases around the world. Due to the high mortality rates and large population of patients, COPD has increasingly become a huge social burden and public health challenge both in China and worldwide [1], [2]. It has been reported that COPD ranked fourth as a leading cause of death in urban areas of China in the year 2008. Additionally, the hospitalization rates for COPD increased from 1.0% in 1998 to 1.6% in 2008 [3]. However, a paucity of biomarkers for COPD has led to delayed diagnosis and interventions for patients with COPD.

COPD is pathophysiologically characterized by persistent airflow limitation and progressive lung function decline [4]. Such airflow obstruction is usually caused by small airway disease (obstructive bronchiolitis) and destruction of lung parenchyma (emphysema). Long-term exposure to noxious particles or gases triggers abnormal inflammatory response in the lung which consequently induces the above pathologic differences and eventually causes COPD [5]. Tobacco smoking is considered to be one of the main risk factors for COPD [6]. Reports from the obstructive lung disease in northern Sweden showed that up to 50% of elderly smokers eventually developed COPD [7]. Apart from smoking, other environmental stimuli, such as air pollution [8] and occupational exposures [9], [10], are increasingly recognized as major risk factors for COPD. Additionally, genetic susceptibility has been shown to be involved in the pathogenesis of COPD. It is well established that α_1_ antitrypsin deficiency is responsible for 1–2% of COPD patients [11]. At the pathological level, inhalation of particles or gases can lead to the infiltration of inflammatory cells and wall thickening in the small airways [12], [13]. Both innate and adaptive immune immunity are involved in enhancing the abnormal inflammatory responses [14], [15], [16]. Reports demonstrated that the population of proinflammatory T-helper-17 cells increases in COPD which thereby causes impaired immune regulation [17], [18]. Whereas oxidative stress produced by reactive oxygen species (ROS) in tobacco smoke is considered to participate in the pathogenesis of COPD [19], [20], the imbalance of proteases and antiproteases is thought to be associated with emphysema [21], [22]. However, the molecular pathogenesis of COPD is complicated, and thus further studies are necessary to characterize the precise underlying mechanisms.

As an important part of systems biology, metabonomics is usually defined as “the quantitative measurement of the dynamic multi-parametric metabolic response of living systems to pathophysiological stimuli or genetic modification” [23]. By measuring all metabolites in a given biological sample, such as a cell, this approach can tell us what indeed happened in that cell and thus improve our understanding of the biological mechanisms of human diseases [24]. Two high-throughput techniques, nuclear magnetic resonance (NMR) spectroscopy and mass spectrometry (MS), are typically employed to investigate the biochemical components of a given sample in metabonomics studies [25]. Due to its high efficient and noninvasive properties, NMR spectroscopy based metabonomics is extensively used to explore the metabolic profiling of biofluids [26], [27], [28], [29]. The metabolic differences in biofluids, such as serum and urine, reflect distinct metabolic processes occurring in the organism. Thus, NMR spectroscopy based serum or urine metabonomics may be employed to determine the diagnosis and prognosis of disease [30], [31].

Recently, several studies have reported the application of NMR spectroscopy based metabonomics in COPD research. It has been shown that NMR spectroscopy based metabolic profiles of exhaled breath condensate could enable discrimination between COPD and healthy subjects [32]. McClay *et al*. found that metabolites trigonelline, hippurate and formate in urine were associated with baseline lung function, whereas no significant associations were found with serum metabolites [33]. Additionally, another report demonstrated that increased protein turnover occurs in all COPD patients with increased protein degradation in individuals with emphysema and cachexia by combining NMR and MS analysis [34].

The main aims of this study were to investigate the metabolic differences between COPD patients and healthy control subjects and to identify potential biomarkers for the diagnosis of COPD. We found that there were substantial metabolic differences between COPD patients and healthy subjects in serum as well as in urine samples. Our results showed the potential applications of this approach as a diagnostic tool for COPD.

## Materials and Methods

### Ethics Statement

All subjects were recruited at The Fourth Affiliated Hospital of China Medical University. Written informed consent was obtained from each subject and the protocol was approved by the Ethics Committee of China Medical University.

### Subjects

The characteristics of the subjects enrolled in this study are summarized in Table 1. A total of 32 patients with COPD (17 female, 15 male, age range 51–79, average age 70) and 21 healthy control subjects (6 female, 15 male, age range 30–85, average age 65) were included in the present study. COPD patients were further classified into four distinct stages according to Global Initiative on Obstructive Lung Disease (GOLD) guidelines as indicated in Table 1. There were 21 smokers and 11 nonsmokers in COPD patients. Among all healthy control subjects 9 were smokers, 12 never smoked. The average smoking histories for COPD patients and controls were 33 and 37 years, respectively. Exercise and diet were not assessed in this study. In order to minimize dietary influence, blood and urine samples were collected from all subjects after overnight fasting. Blood samples were collected from each subject into sodium-heparin tubes and then centrifuged at 1,500 × g, 4°C, for 15 min. Approximately 1 mL plasma aliquots were transferred into sterile cryovials and stored at -80°C. Morning urine samples were obtained from all subjects after overnight fasting. Aliquots of approximately 4 mL were collected and stored at -80°C.

**Table 1:** Characteristics of COPD Patients and Healthy Controls^a^.

	COPD patients	Healthy controls
No. of subjects	32	21
Gender	M (15), F (17)	M (15), F (6)
Average age (range)	71 (51–79)	63 (30–85)
Smoking habits	Smokers 21	Smokers 9
	Nonsmokers 11	Nonsmokers 12
FEV_1%_	24±35	155±69
FVC %	39±55	199±54
GOLD stage	I 1	
	II 6	
	III 3	
	IV 22	

a FEV_1_, forced expiratory volume in 1 s; FVC, functional vital capacity. Date for FEV_1%_ and FVC % are expressed as mean ± SD.

### Samples Preparation and NMR Measurements

At the time of NMR analysis, plasma aliquots were thawed at room temperature and then the plasma (400 µL) was mixed with phosphate buffered saline (PBS) (0.6 M, 30 µL) and D_2_O (170 µL) (to provide field frequency lock). The homogenized mixture was subsequently centrifuged at 12,000 rpm, 4°C, for 10 min. The supernatant (500 µL) for each sample was transferred into a 5 mm NMR tube. Similarly, all urine samples were thawed and prepared by mixing urine (500 µL) with PBS (1 M, pH 7.4, 100 µL) (containing 0.1% w/v TSP). The mixture was homogenized and maintained at room temperature for 5 min. After centrifugation at 12,000 rpm, 4°C, for 10 min, the supernatant (500 µL) for each sample was transferred into a 5 mm NMR tube. All ^1^H NMR spectra were acquired on a Varian Inova 600 MHz spectrometer (Varian Inc., Palo Alto, CA, USA) operating at 599.92 MHz for ^1^H observation, at 298 K. For serum samples, one-dimensional NMR spectra were recorded using a standard Carr-Purcell-Meiboom-Gill (CPMG) pulse sequence to suppress the spectral interferences from macromolecules and to improve visualization of low molecular weight metabolites. Spectra were acquired with a spectral width of 8000.0 Hz and an acquisition time of 1.0 s. Relaxation delay was set at 2.1 s. A total echo time of 100 ms was used in the CPMG sequence. For urine samples, all ^1^H NMR spectra were collected using a standard 1D nuclear overhauser enhancement spectroscopy (NOESY)-presaturation pulse sequence. Typically, 64 scans were accumulated with a spectral width of 8384.9 Hz, an acquisition time of 0.9541 s and a relaxation delay of 2.1 s.

### Data Processing

The obtained spectra of serum and urine samples were Fourier transformed with TopSpin software version 3.0 (Bruker Biospin, Germany). All 1D spectra were processed with a line broadening of 1 Hz, manually phased and baseline-corrected. The detected signals were assigned based on matching the obtained NMR data to reference spectra in the Human Metabolome Database version 3.0, as well as other existing databases and previous literature reports [35], [36], [37]. The NMR spectra of serum were referenced and scaled to the lactate signal at 1.33 ppm. The integration was performed over 9.0–0.5 ppm region with the bucket width set to 0.002 ppm. The regions corresponding to the spectrum signal of residual water, complexes and ethanol (3.69–3.58, 3.30–3.05, 2.73–2.50 and 1.22–1.16 ppm) were excluded. The NMR spectra of urine were referenced and scaled to the TSP signal at 0 ppm. The spectra in the region 10.0–0.5 ppm were integrated with the bucket width set to 0.005 ppm, leaving out the region 6.3–5.5 and 5.2–4.4 ppm, which included residual water and urea, respectively. Finally, all spectra were normalized to the total spectral area.

### Multivariate Statistical Analysis

A total of 53 spectra which obtained from 32 COPD patients and 21 healthy controls were subjected to multivariate statistical analysis. After normalization, principal component analysis (PCA), partial least squares discriminant analysis (PLS-DA) and orthogonal projections to latent structures (OPLS)-DA were performed using SIMCA-P+ software version 11.0 (Umetrics, Umea, Sweden). The unsupervised PCA was first carried out to detect intrinsic clusters and possible outliers within the data set using mean centered data. Subsequently, supervised PLS-DA modeling was applied to improve class discrimination between COPD patients and healthy controls using unit variance scaling and mean center scaling methods for serum and urine spectra data, respectively. The quality of the models was assessed by a 10-fold cross-validation method. The obtained parameters R^2^X, which stands for the total explained variation of the model, and Q^2^, which represents the predictability of the model, were further used to confirm the validity of these models. Finally, these PLS-DA models were validated by a 100-times permutation test. To maximize the class discrimination, OPLS-DA was performed using unit variance scaling method. OPLS-DA is an extension of PLS-DA featuring an integrated Orthogonal Signal Correction (OSC) filter to remove variability not relevant to class separation [28]. The unit variance scaling gives the same weight to all the spectral variables regardless of the peak heights. It is benefit for the detection of low concentration metabolites that contribute to the discrimination between clusters. The metabolites responsible for discriminating COPD patients from healthy controls were indicated in the coefficient plots which were calculated by back transformation of the loadings. The significance test of the Pearson's product-moment correlation coefficient was used to determine metabolites with significant difference between COPD patients and healthy controls. The correlation coefficient of |r|>0.400 was used as the cut-off value for significance based on discrimination significance at *P*<0.05. Q^2^>0.1 was considered acceptable for PLS-DA and OPLS-DA models.

## Results

### Determination of Metabolic Differences in Serum and Urine According to ^1^H NMR Spectroscopy

**Figure 1:**
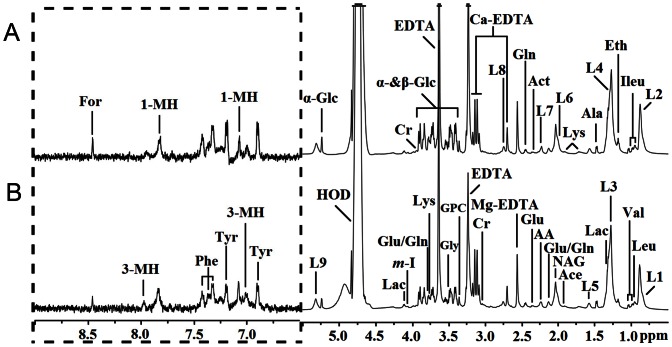
Representative CPMG ^1^H NMR spectra of serum obtained from (A) controls and (B) COPD patients. The region of δ 6.5–9.0 (in the dashed box) was magnified 16 times compared with corresponding region of δ 0.5–5.5 for the purpose of clarity. Keys: 1-MH: 1-Methylhistidine; 3-MH: 3-Methylhistidine; AA: Acetoacetate; Ace: Acetate; Act: Acetone; Ala: Alanine; Cr: Creatine; Eth: Ethanol; For: Formate; Glc: Glucose; Gln: Glutamine; Glu: Glutamate; Gly: Glycine; GPC: Glycerolphosphocholine; Ileu: Isoleucine; L1: HDL, CH3-(CH2)n-; L2: VLDL, CH3-(CH2)n-; L3: HDL, CH3-(CH2)n-; L4: VLDL, CH3-(CH2)n-; L5: VLDL, -CH2-CH2-C = O; L6: Lipid, -CH2-CH = CH-; L7: Lipid, -CH2-CH = CH-; L8: Lipid, -CH2-C = O; L9: Lipid, = CH-CH2-CH = ; L10: Lipid, -CH = CH-; Lac: Lactate; Leu: Leucine; Lys: Lysine; m-I: myo-Inositol; NAG: N-acetyl glycoprotein signals; Phe: Phenylalanine; Tyr: Tyrosine; Val: Valine.

**Figure 2:**
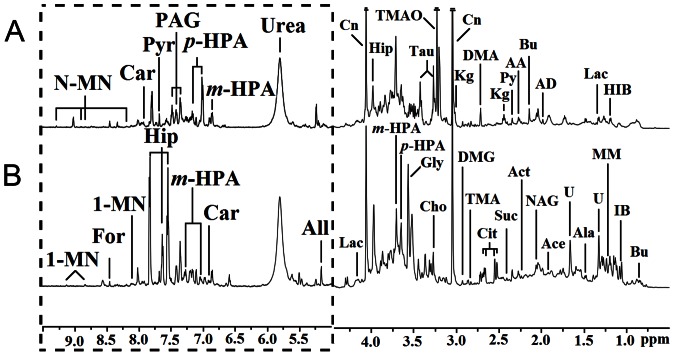
Representative NOESYPR1D ^1^H NMR spectra of urine obtained from (A) controls and (B) COPD patients. The region of δ 5.0–9.5 (in the dashed box) was magnified 16 times compared with corresponding region of δ 0.5–4.5 for the purpose of clarity. Keys: 1-MN: 1-Methylnicotinamide; Ace: Acetate; Act: Acetone; Ala: Alanine; All: Allantoin; AD: Acetamide; Bu: Butyrate; Car: Carnosine; Cit: Citrate; Cho: Choline; Cn: Creatinine; DMA: Dimethylamine; DMG: Dimethylglycine; For: Formate; Gly: Glycine; Hip: Hippurate; IB: Isobutyrate; Kg: α-Ketoglutarate; Lac: Lactate; MM: Methylmalonate; N-MN: N-Methylnicotinamide; NAG: N-Acetylglutamate; PAG: Phenylacetyglycine; p-HPA: p-Hydroxyphenylacetate; Py: Pyruvate; Pyr: Pyridoxine; Suc: Succinate; Tau: Taurine; TMA: Trimethylamine; TMAO: Trimethylamine-N-Oxide; U: Unknown; α-HB: α-Hydroxybutyrate; α-HIB: α-Hydrxoy-isobutyrate.

Serum and urine samples were collected from COPD patients (n = 32) and healthy controls (n = 21), respectively. Representative 600 MHz ^1^H NMR spectra of serum and urine samples from a COPD patient and a healthy subject are shown in Figure 1 and 2, respectively. Abundant endogenous metabolites were detected both in serum and urine according to these spectral data. The dominant metabolites detected in serum were lipoproteins and several amino acids (e.g., alanine, lysine, glutamine, glycine, histidine) (Figure 1). The most intense signals in urine arose from glycine, creatinine, citrate, α-ketoglutarate, trimethylamine-N-oxide and p-hydroxyphenylacetate (Figure 2). Due to the high interindividual variability and large complexity, it’s impossible to get consistent comparison results across all the subjects. Thus, the NMR spectra of serum and urine samples were subjected to multivariate statistical data analysis in order to identify the metabolic differences between COPD patients and healthy controls.

### Discrimination between COPD Patients and Controls

**Figure 3:**
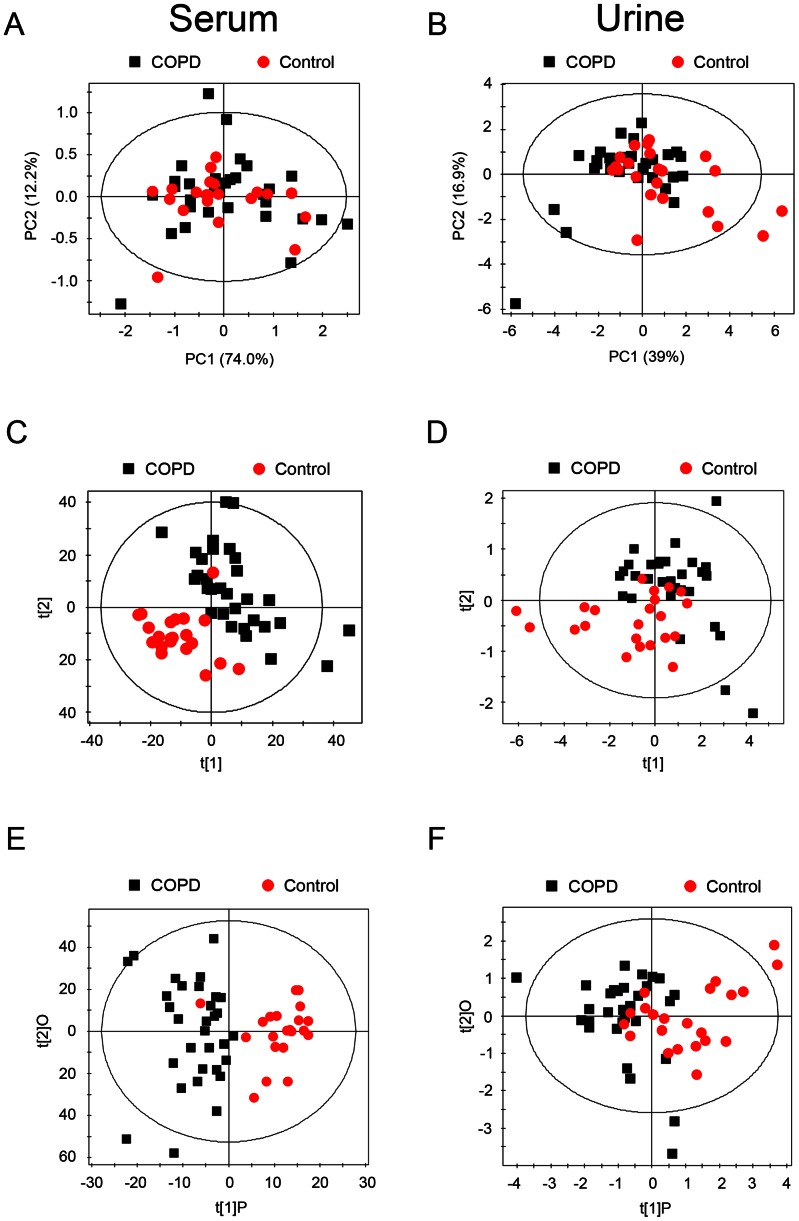
Multivariate data analysis of ^1^H NMR spectra obtained from COPD patients and healthy controls. Scores scatter plots generated from applying (A) PCA, (C) PLS-DA and (E) OPLS-DA to the ^1^H NMR spectra of serum. The corresponding scores plots derived from ^1^H NMR spectra of urine are shown in (B), (D) and (F), respectively.

To generate an overview of the variations between COPD patients and healthy control subjects, PCA was first performed based on the normalized NMR spectral data obtained from serum and urine samples. The first and second principal components (PC1 and PC2) were calculated for the models of comparing COPD patients with healthy controls. The first two PCs account for a total of 86.2% and 55.9% of variance for serum and urine samples, respectively. According to the established PCA models, one urine sample obtained from a COPD patient was found to be an outlier and consequently removed for further investigation (data not shown). The final PCA results were illustrated in Figure 3. While there was significant superimposition of COPD patients and healthy controls, a trend for unsupervised separation between these two groups was found in the PC1 *vs.* PC2 scores scatter plots (Figure 3A and 3B), particularly for urinary metabolic profiles (Figure 3B).

**Figure 4:**
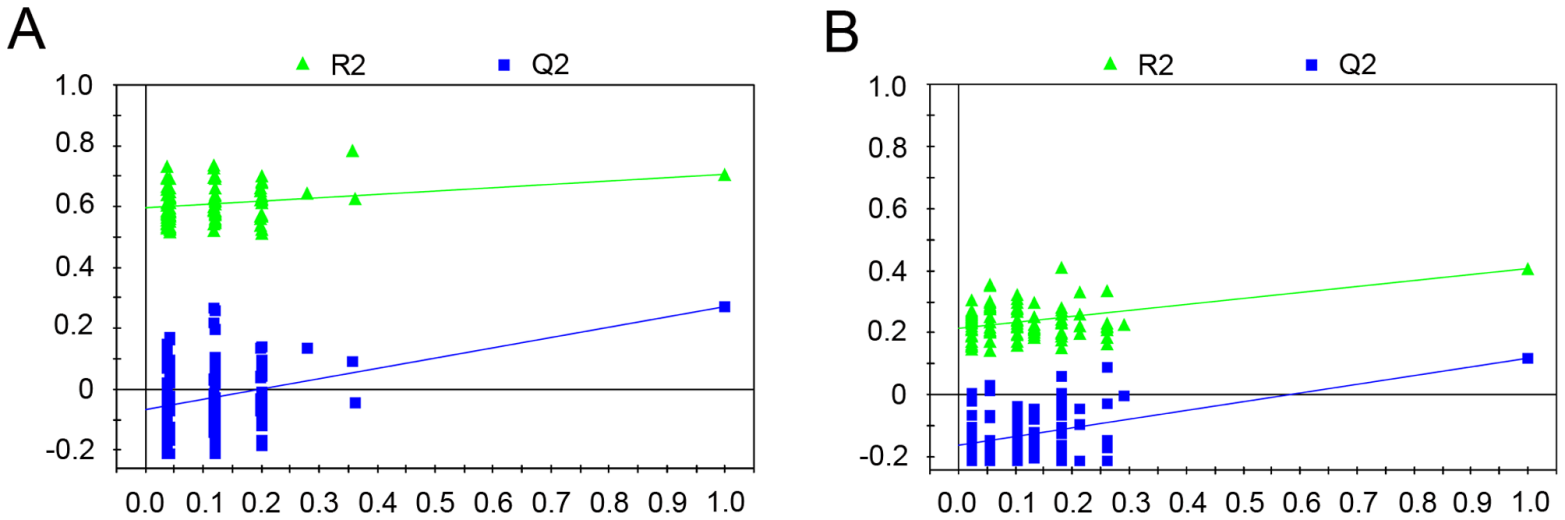
Plots of permutation test for PLS-DA modes. A 100 random permutation test for PLS-DA modes generated from (A) serum or (B) urinary profiles of COPD patients and healthy controls.

In order to get a better separation, PLS-DA was applied in our study. The quality of the models was assessed by a 10-fold cross-validation method (i.e., constructing models repeatedly by leaving out one-tenth of the samples and predicting them back into the model) [38]. Subsequently, the obtained parameters R^2^X, which stands for the total explained variation of the model, and Q^2^, which represents the predictability of the model, were used to describe the quality of these models. As shown in Figure 3, a reasonably good separation was achieved in serum (R^2^X = 20.5%, Q^2^ = 0.272) (Figure 3C) as well as in urine (Figure 3D) (R^2^X = 48.6%, Q^2^ = 0.120). The sensitivity and specificity of PLS-DA model generated from serum profiles were 90% and 86.95%, respectively, and the classification rate was 88.72% (Table 2). Likewise, high sensitivity (87.74%), specificity (89.45%) and classification rate (88.45%) were obtained for PLS-DA model based on NMR spectral data collected from urine samples (Table 2). Furthermore, the robustness of these PLS-DA classification models was assessed by a 100-times permutation test. The R^2^ and Q^2^ values derived from the permuted data were lower than the original ones and the regression of Q^2^ lines intersected at below zero, indicating the validation of these PLS-DA models (Figure 4A and 4B).

**Table 2:** Prediction Results of PLS-DA Models Based on ^1^H NMR Spectra Obtained from COPD Patients and Healthy Controls^a^.

	No. of subjects	Sensitivity (%)	Specificity (%)	Classification rate (%)
Serum	COPD (n = 26) *vs.* Control (n = 20)	90	86.95	88.72
Urine	COPD (n = 31) *vs.* Control (n = 21)	87.74	89.45	88.45

a Sensitivity and specificity values are based on 100-fold-cross-validation. Sensitivity was determined from the ratio of true positives (COPD samples correctly predicted) to total number of modeled COPD spectra, whereas specificity was calculated from the ratio of true negatives (control samples correctly predicted) to total number of modeled control spectra. Classification rate was expressed as the ratio of total number of samples correctly classified to total number of samples predicted.

Therefore, the results of multivariate statistical analysis demonstrated that there were significant metabolic differences both in serum and urine as comparing COPD patients with healthy controls.

### Identification of Discriminatory Metabolites

**Figure 5:**
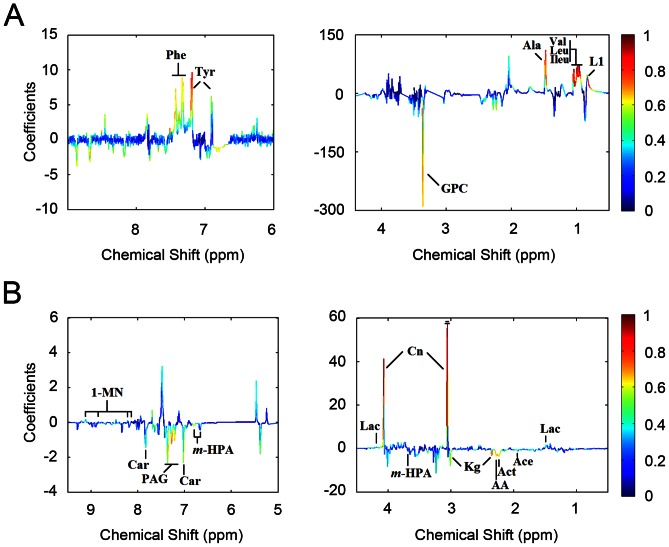
Coefficient loading plots obtained from serum and urine. Coefficient loading plots calculated from OPLS-DA modeling of (A) serum and (B) urine. Peaks in the positive direction indicate metabolites with increased expression levels in healthy controls, whereas the negative direction peaks denote metabolites display enhanced expression levels in COPD patients. The color scaling maps on the right-hand side of each coefficient loading plot represent the contribution of metabolites in discriminating COPD patients from healthy control subjects. Keys of the assignments were shown in Figure 1 and 2, respectively.

To identify the main metabolites responsible for discriminating COPD patients from healthy control subjects, OPLS-DA was carried out with a unit variance scaling strategy to further analyze the metabolite profiles obtained from serum and urine samples. The OPLS-DA score plots and corresponding coefficient loading plots based on the collected NMR data are presented in Figure 3 and Figure 5, respectively. The separation between COPD patients and healthy controls was further improved both for serum (R^2^X = 20.5%, Q^2^ = 0.265) (Figure 3E) and urine (R^2^X = 48.6%, Q^2^ = 0.154) (Figure 3F) samples after OPLS-DA was employed. Moreover, the color-coded coefficient loading plots revealed more detailed information about metabolic differences between COPD patients and healthy controls (Figure 5). Here, the direction of the signals associates with the relative variations of metabolites in COPD patients compared to the healthy controls. For instance, peaks in the positive direction indicate metabolites that are more abundant in healthy controls, whereas the negative direction peaks denote metabolites significantly higher in COPD patients. The color scaling maps on the right-hand side of each coefficient loading plot represent the contribution of metabolites in discriminating COPD patients from healthy control subjects. For example, red indicates a more significant contribution to the separation between these two groups than blue. According to the loading plots, increased expression levels of phenylalanine, tyrosine, alanine, valine, leucine, isoleucine and high density lipoprotein (HDL) were found in the serum of healthy controls (Figure 5A). However, only glycerolphosphocholine (GPC) was elevated in COPD patients (Figure 5A). Interestingly, there are more significant metabolic differences in urine between COPD patients and healthy controls. As shown in Figure 5B, metabolites, including 1-methylnicotinamide (1-MN), creatinine and lactate exhibited an elevated expression level in healthy controls. On the other hand, enhanced expression levels of carnosine, phenylacetyglycine, pyruvate, m-hydroxyphenylacetate, α-ketoglutarate, acetate, acetoacetate and acetone were found in the urine of COPD patients relative to healthy controls (Figure 5B). The coefficients indicating the significance of the metabolites contributing to the separation in serum and urine were summarized in Tables 3 and 4, respectively. Here, the coefficient of 0.4 was used as the cut-off value which was calculated based on discrimination significance at the level of 0.05.

**Table 3:** OPLS-DA Coefficients Derived from the NMR Data of Metabolites in Serum Obtained from COPD Patients and Healthy Controls.

Metabolites	r^a^
	Control *vs.* COPD
Alanine: 1.48(d^b^)	0.516
GPC: 3.36(s)	−0.449
Isoleucine: 0.94(t), 1.01(d)	0.538
L1: HDL, CH3-(CH2)n-	0.508
Leucine: 0.96(d)	0.598

a Correlation coefficients, positive and negative signs indicate positive and negative correlation in the concentrations, respectively. The correlation coefficient of |r|>0.400 was used as the cut-off value for the statistical significance based on the discrimination significance at the level of *p*-value = 0.05.

b Multiplicity: s, singlet; d, doublet; t, triplet; q, quartet; dd, doublet of doublets; m, multiplet.

**Table 4:** OPLS-DA Coefficients Derived from the NMR Data of Metabolites in Urine Obtained from COPD Patients and Healthy Controls.

Metabolites	r^a^
	Control *vs.* COPD
1-MN: 8.02(t^b^), 8.09(s), 8.85(d), 9.13(s)	0.515
Acetate: 1.93(s)	−0.421
Acetoacetate: 2.28(s)	−0.603
Acetone: 2.23(s)	−0.457
Carnosine: 7.03(s), 7.86(s)	−0.461
Creatinine: 3.05(s), 4.06(s)	0.763
Lactate: 1.33(d), 4.13(q)	0.465
m-Hydroxyphenylacetate: 3.72(s), 6.67(d),6.86(d), 7.28(t)	−0.575
Phenylacetyglycine: 7.32(d), 7.36(t), 7.42(dd)	−0.515
Pyruvate: 2.35(s)	−0.611
α-Ketoglutarate: 2.45(t), 3.01(t)	−0.460

a Correlation coefficients, positive and negative signs indicate positive and negative correlation in the concentrations, respectively. The correlation coefficient of |r|>0.400 was used as the cut-off value for the statistical significance based on the discrimination significance at the level of *p*-value = 0.05.

b Multiplicity: s, singlet; d, doublet; t, triplet; q, quartet; dd, doublet of doublets; m, multiplet.

## Discussion

In the present study, ^1^H NMR spectroscopy based metabonomics analyses were utilized to explore the metabolic differences between COPD patients and healthy subjects and to identify characteristic metabolites in COPD patients.

The obtained serum and urine NMR spectral data were subjected to multivariate statistical data analysis. Intriguingly, the analysis showed that metabolic differences were more prominent in urine than in serum which is in accordance with previously reported work [33]. In serum, decreased HDL, alanine, isoleucine, leucine, phenylalanine, tyrosine, valine, and increased GPC were found in COPD patients. Regarding to the serum concentration of HDL in COPD patients, conflicting results have been reported [39], [40], [41]. HDL particles are capable of transporting cholesterol from within artery atheroma to the liver for excretion. Lower serum level of HDL may increase the risk of cardiovascular diseases [42]. Decreased HDL may account for a 2- to 3-fold increased risk of cardiovascular disease among COPD patients [40], [43]. The reduction in HDL level has also been found in lung cancer [28]. It is well documented that amino acids are not only nutrition but also play vital roles in regulating key metabolic pathways, thus it’s crucial to keep the metabolic balance of amino acids for health [44]. In line with two recent reports, perturbation in amino acid metabolism has also been observed in serum samples from COPD patients in our present study [34], [45]. Among these, the most attractive amino acids are valine, isoleucine and leucine which all belong to branched-chain amino acids (BCAAs). Actually, decreased BCAAs have been found in the published literatures regarding COPD patients [46], [47], [48]. BCAAs play a key role in regulating glucose homeostasis and protein turnover [34]. Decreased BCAAs in COPD patients is possible due to enhanced gluconeogenesis under hypoxia conditions caused by limited air exchange. In addition, anorexic effects of COPD, such as low food intake, may contribute to the decline of BCAAs in serum. The low level of BCAAs in COPD patients may also be related to increased risk of coronary heart disease [49], [50]. However, it should be noted that decreased phenylalanine was observed in the present study which differs from earlier reports [34], [45]. One possible explanation for this disagreement may arise from different lifestyle and genetic background, since these studies have been conducted with European subjects.

Unlike in serum, there were more significant metabolic differences in urine samples. It was found that 1-MN, creatinine and lactate were reduced in COPD patients relative to healthy subjects. 1-MN, a methylation product of nicotinamide, has been shown to exert anti-inflammatory properties and scavenge oxygen radicals [51], [52]. Decreased 1-MN in COPD patients indicates impairment of the nicotinate and nicotinamide metabolism which has also been observed in urine samples from lung cancer [28]. Creatinine is one of the most abundant metabolites in urine and its levels are affected by several factors, such as dietary habit and muscle mass. However, these factors were not controlled in our research. Thus, careful considerations should be taken into account in interpreting the results. Reduced creatinine has also been found in liver cancers [53].

In addition to the above-mentioned metabolite differences, elevated levels of acetate, acetoacetate, acetone, carnosine, m-hydroxyphenylacetate, phenylacetyglycine, pyruvate and α-ketoglutarate have been discovered in urine from COPD patients compared to healthy subjects. Acetate is the final product of lipid metabolism and it can be catalyzed to acetyl-coenzyme A (acetyl-CoA) by acetyl-CoA synthetase [54]. Therefore, its increase may reflect an accelerated lipid catabolism in order to satisfy the energy requirements result from the poor nutritional status generally associated with COPD patients. Ketone bodies, including acetoacetate and acetone, are produced from acetyl-CoA when energy is obtained from breaking down fatty acids due to lack of carbohydrates. The observed increases in acetoacetate and acetone may indicate the utilization of storage lipids as an alternative energy resource for COPD patients. Another alternative metabolite, carnosine, has been proved to possess a number of antioxidant properties [55], [56]. Hence, we speculate that enhanced expression level of carnosine may be beneficial for COPD patients to scavenge ROS and α, β-unsaturated aldehydes generated by lipid peroxidation during oxidative stress. Besides, it is of great interest to note that m-hydroxyphenylacetate is higher in COPD patients compared to healthy subjects. To our knowledge, this is the first report regarding the difference of such metabolite between COPD patients and healthy controls. Therefore, no explanation for the increase of this metabolite is proposed at present. Regarding phenylacetyglycine, increased excretion has also been reported in lung cancer as well as model rats of colorectal cancer, possibly relating to gut microflora metabolism [28], [57]. Finally, other metabolites found to be increased in COPD patients are pyruvate and α-ketoglutarate. While pyruvate is the end product of glycolysis, α-ketoglutarate is an intermediate in the tricarboxylic acid (TCA) cycle. Depending on the availability of oxygen, pyruvate can be further degraded to acetyl-CoA which enters the TCA cycle or to lactate in animals and ethanol in plants and microorganisms [58]. Consistent with our preliminary result, elevated expression level of pyruvate has also been found in exhaled breath condensate samples from COPD patients as well as in serum from lung cancer [29], [32]. However, it is not clear yet which downstream pathway the increased pyruvate will enter. Recently, Kao *et al*. demonstrated that increased production and oxidation of pyruvate are found in COPD subjects compared to controls and the majority of this pyruvate is then oxidized via the TCA cycle rather than being disposed via non-oxidative pathways to lactate [59]. Therefore, this study may provide clues to address the above issues. Additionally, the enhanced level of α-ketoglutarate suggests a possible higher demand on the TCA cycle for COPD patients.

In spite of the consistent metabolic differences between COPD patients and healthy subjects, it should be noted that there are still some limitations in the present study. Although all blood and urine samples were collected after overnight fasting, in order to minimize the dietary influence, we cannot rule out this possibility completely. Because the metabolism of some food constituents can affect blood and urine compositions even several hours after intake. However, as none of the subjects were under the same diet, it is unlikely that diet would account for the consistent metabolic differences found between these two groups. Another aspect to note is the relatively small amount of subjects adopted in the present study. The metabolic profile of an individual reflects complicated interactions between genetic and environmental factors. COPD is such a complex disease which involves genetic susceptibility and long-term exposure to noxious particles or gases. Consequently, future studies with a large number of subjects are necessary so as to obtain robust conclusions. Moreover, other possible cofounders including age, gender, body mass index and smoking should be assessed in the future, as these might also influence the metabolic variations between COPD patients and controls.

In conclusion, the present study revealed that ^1^H NMR spectroscopy combined with multivariate statistical analysis detected consistent metabolic differences between COPD patients and healthy controls in serum as well as in urine samples. Our findings reinforce the possibility for the potential applications of this approach in early diagnosis and patient management. Future prospective studies with more abundant subjects and detailed assessment of the possible cofounders as mentioned above may achieve solid conclusions and provide new insights to understand the pathogenesis of COPD.
